# The Impact of Health Literacy and Religiosity on Attitudes Towards Physician-Assisted Death

**DOI:** 10.1093/geroni/igab046.3340

**Published:** 2021-12-17

**Authors:** Amy Albright, Joshua Tutek, Rebecca Allen

**Affiliations:** 1 VA Maine HCS, Gorham, Maine, United States; 2 Stanford University, Stanford University School of Medicine, California, United States; 3 University of Alabama, Tuscaloosa, Alabama, United States

## Abstract

The aim of the current study was to examine the relationship between functional health literacy and religiosity regarding attitudes towards physician-assisted death (PAD). Of participants, the majority were female (62.6%) and non-Hispanic White (79.6%), and ages ranged from 19 to 83 (M = 37.81, SD = 12.55). As measured by the Newest Vital Sign, 82.6% (n = 219) of individuals within the current sample had adequate functional health literacy, while 10.6% (n = 28) scored within the “possibly limited” range, and 6.8% (n = 18) scored within the “highly limited” range. There was a positive association between religiosity and age (r = .21, p < .001), and older participants were more likely to endorse religious beliefs and/or activities. There was a significant association between greater acceptance of attitudes towards PAD and functional health literacy (r = .17, p < .01), indicating that those with higher health literacy have more positive attitudes towards PAD. There was no significant association between attitudes towards PAD and age (r = -.02, p > .05) or education (r = -.05, p > .05). Similarly, attitudes did not differ by gender (t (256) = -.66, p > .05) or by race/ethnicity, (F(5, 253) = .73, p > .05). Of note, functional health literacy may be particularly important to monitor in this context, as several studies (i.e., Kobayashi et al., 2015) have shown that health literacy may decrease with mild cognitive impairment and may therefore provide important information regarding older adults with this condition.

